# Oral Phenytoin Toxicity Causing Sinus Arrest: A Case Report

**DOI:** 10.1155/2014/851767

**Published:** 2014-09-11

**Authors:** Ravi K. Thimmisetty, Janardhana Rao Gorthi, Mahmoud Abu Hazeem

**Affiliations:** ^1^Department of Internal Medicine, Creighton University Medical Center, 601 N 30th Street, Omaha, NE 68131-0216, USA; ^2^Division of Cardiology, University of Minnesota, Mayo Mail Code 508, 420 Delaware Street SE, Minneapolis, MN 55455, USA

## Abstract

We present a case of sinus node arrest leading to symptomatic junctional bradycardia from oral phenytoin toxicity, which is a rare presentation. Our patient had no prior cardiac history and was on phenytoin therapy for seizure disorder. Although bradycardia is more commonly associated with intravenous phenytoin and there were few case reports of bradycardia with oral phenytoin reported, the literature is limited. In this case report, we also reviewed the pathophysiology of phenytoin-induced cardiac toxicity.

## 1. Introduction

Phenytoin is a frequently used antiepileptic medication for the treatment of seizure disorder. The common side effects include nausea, rash, gingival hypertrophy, osteomalacia, and confusion. The potential to cause cardiac rhythm disturbances, hypotension, and cardiac arrest is rarely recognized.

## 2. Case Report

A 50-year-old woman presented to the emergency room with a nonproductive cough and dyspnea for four days. Past medical history included cerebral palsy, epilepsy, and quadriparesis. Home medications were phenytoin 150 mg bid for 6 months, levetiracetam 2 gm bid, valproic acid 1.5 gm bid, phenobarbital 32.4 mg bid, and lacosamide 200 mg bid. There is no family history of premature coronary artery disease or sudden cardiac death.

Vital signs on admission were a temperature of 36.7°C, blood pressure of 98/60 mm/Hg, heart rate of 38 beats per minute, and respiratory rate of 15 breaths per minute, and oxygen saturation was 97% on two liters of oxygen via nasal cannula. Heart exam revealed bradycardia with S1, S2 without murmurs, rubs, or gallops. The remaining physical exam was normal.

**Figure 1:**
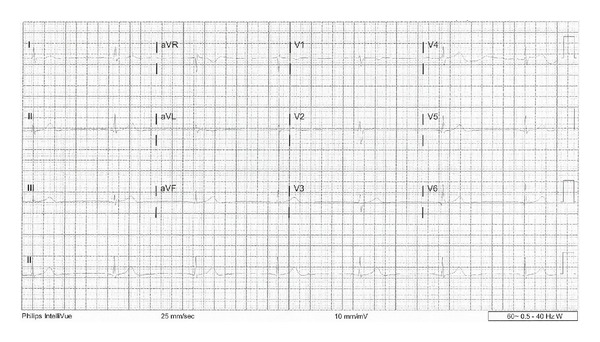
Junctional bradycardia. Electrocardiogram (ECG) recording speed of 25 mm/s.

**Figure 2:**
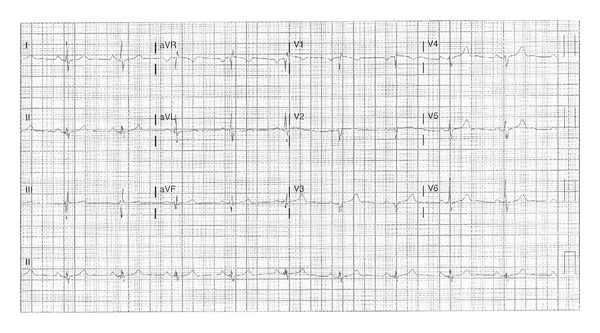
Baseline ECG. ECG recording speed of 25 mm/s.

The patient received intravenous normal saline for symptomatic hypotension. An initial electrocardiogram (ECG) revealed junctional bradycardia with a heart rate of 35 beats per minute, PR interval which is not measurable, corrected QT interval of 386 milliseconds, QRS interval of 90 milliseconds, and QTc interval of 390 milliseconds (Figure 1). Baseline ECG was normal sinus rhythm with a normal rate (Figure 2). Laboratory findings on admission included normal complete blood counts and a comprehensive metabolic panel, including electrolytes (serum sodium is 133 meq/L, potassium is 4.4 meq/L, magnesium is 1.9 mg/dL, and calcium is 9.3 mg/dL).

Coagulation parameters were within normal (prothrombin time/internationalized normalized ratio, partial thromboplastin time). Brain natriuretic peptide (BNP) was slightly elevated at 394 picogram/mL (normal range is <100 picogram/mL), thyroid stimulating hormone (TSH) was 3.5 micro IU/mL (normal range is 0.5–5.5), cortisol level was 26 microgram/mL (normal range is 7–28), and serum valproate level was 58.7 (normal range is 50–100). The serum phenytoin level was elevated at 36.4 (normal range is 10–20 microgram/dL). Transthoracic echocardiogram (TTE) showed normal ejection fraction and no regional wall motion abnormalities. Chest X-ray was normal. Blood cultures revealed no growth.

**Figure 3:**
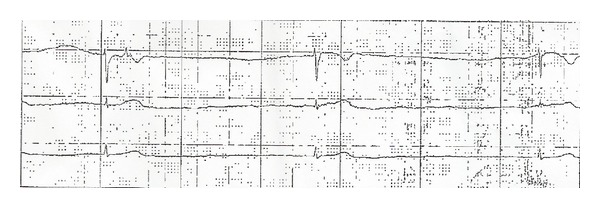
Telemetry rhythm strip showing sinus arrest with junctional escape rhythm at rate of 22 beats per minute.

We held phenytoin, owing to high serum levels, and placed atropine and pacer pads at the bedside. A few hours after admission, the patient became unresponsive and telemetry revealed severe junctional bradycardia and asystole (Figure 3). Cardiopulmonary resuscitation was initiated immediately, with a return of spontaneous circulation (ROSC) achieved in 2 minutes. Blood pressures at ROSC were 90/60 mmHg and bradycardia persisted with heart rates around 30–35. The patient was intubated and started on dopamine and epinephrine infusions.

**Figure 4:**
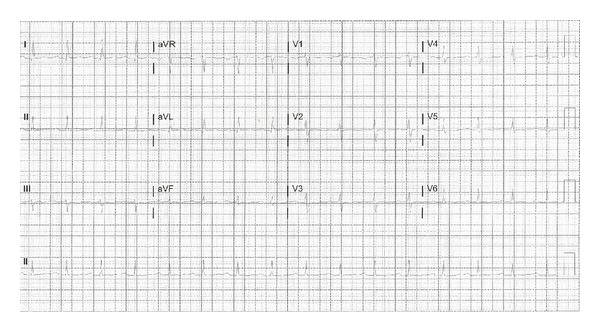
The ECG shows normal sinus rhythm without any pacer spikes. ECG recording speed of 25 mm/s.

**Figure 5:**
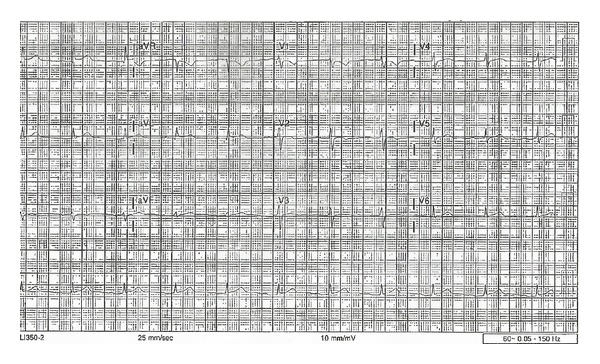
The ECG shows normal sinus rhythm. ECG recording speed of 25 mm/s.

A temporary transvenous pacemaker was placed for symptomatic bradycardia and hypotension via a femoral approach. Laboratory findings drawn at this time revealed normal electrolytes (serum sodium is 135 meq/L, calcium is 8.0 mg/dL, magnesium is 2.0 mg/dL, and potassium is 4.2 meq/L) and all the three sets of successive cardiac enzymes were negative. The patient subsequently improved hemodynamically, resulting in discontinuation of vasopressors. Repeated serum phenytoin level is 32 (normal range is 10–20 microgram/dL). After 24 hours of pacemaker placement, the ECG showed normal sinus rhythm with a normal rate with no pacer spikes (Figure 4). The patient was extubated and the pacemaker was removed. The patient was started on a new antiseizure medication, clobazam, as per neurology recommendations, and electroencephalogram revealed no seizure activity. The patient was monitored for another 24 hours after removal of the pacemaker. We did another ECG after removal of pacemaker which revealed normal sinus rhythm with a heart rate of 65 beats per minute, PR interval of 201 milliseconds, QRS interval of 108 milliseconds, and QT interval of 387 milliseconds (Figure 5). There were no more episodes of abnormal rhythms on telemetry and she was successfully discharged to home.

## 3. Discussion

The episodes of sinus arrest that developed in this patient could be from several causes. We ruled out the probable cardiac causes, such as ischemic heart disease, heart failure, cardiomyopathy, or infection (myocarditis, rheumatic) based on the fact that our patient had no cardiac risk factors or prior cardiac history, negative cardiac enzymes, normal baseline ECG, normal TTE, and normal blood cultures. There was also no evidence of direct carotid body stimulation, which can induce blocks.

Several other causes of sinus node arrest include hyperkalemia or hypokalemia, azotemia, endocrine abnormalities (hypothyroidism, adrenal insufficiency), and hypothermia. These were ruled out because the patient's electrolytes were completely normal (potassium, magnesium), she had normal renal function with no evidence of azotemia, and she had normal thyroid and adrenal function. Medications (e.g., diltiazem, verapamil, beta blockers, digoxin, amiodarone, clonidine, quinidine, procainamide, and disopyramide) can cause sinus node arrest, but our patient was not taking any of them. Temporal lobe epilepsy, frontal lobe epilepsy, and seizures associated with left insular lesions have been shown to be associated with bradycardia and, rarely, asystole, but our patient had normal EEG so these were also excluded [1]. There were no laboratory findings or clinical evidence of adrenal insufficiency in our patient.

Hypothermia was excluded because the patient's temperature during hospitalization was 36.7°C. The patient lives with family. She does have pill organizer which was monitored by her father, so we do not think there is a chance of drug overdose or self-harm.

Excluding all other causes of junctional bradycardia and observing the temporal relation of sinus node arrest to toxic levels of phenytoin, it was concluded that oral phenytoin caused this event. Phenytoin toxicity can be definitely proved, if the adverse effect was observed after reintroduction of phenytoin but, presumably, this would be infeasible in view of the potential severity and life-threatening nature of the adverse effect.

## 4. Pathophysiology

Phenytoin is commonly prescribed antiepileptic drug. In 1938, oral phenytoin (diphenylhydantoin) was introduced as an anticonvulsant; the first case of bradycardia associated with its use was described the following year [2]. The narrow therapeutic index, the wide interindividual variability in the rate of phenytoin metabolism and clearance, and the saturation (zero-order) pharmacokinetics of phenytoin are responsible for the observed dose-related toxicity [3].

Phenytoin is a class IB antiarrhythmic agent and has different effects on cardiac tissue. Both human and animal studies have proven that phenytoin's action can be seen in atrial, ventricular, and AV nodes [4].

This drug can shorten or lengthen the refractory period of atrial tissue to a variable degree. Phenytoin can enhance or delay the AV nodal conduction, leading to a potentiality for tachy-brady arrhythmias. Phenytoin shortens the relative refractory period and suppresses automaticity in ventricular muscle. It does not have much effect on the effective refractory period of the His-Purkinje system [4]. Phenytoin can inhibit cardiac sodium channels and inhibit phase 0 inward intracellular sodium currents, resulting in slowing of conduction and widening of the QRS complex. Phenytoin depresses ventricular automaticity and enhances AV conduction while having little or no effect on interventricular conduction or sinus rate. This property is used for treatment in digitalis toxicity associated with ventricular arrhythmias [5]. Several reports of worsening AV nodal conduction have been reported with the use of intravenous phenytoin in critically ill patients [6, 7]. Due to this known side effect profile, monitoring blood pressure and pulse every 15 minutes for one hour during the initial intravenous administration of phenytoin is recommended. One study demonstrated that 57 patients admitted with oral phenytoin overdose with serum concentrations as high as 56 mcg/mL experienced no significant cardiovascular arrhythmias or complications [8]. There are few case reports of death or cardiovascular toxicity reported with oral phenytoin overdose [9]. Most practitioners are not aware of this rare toxicity of phenytoin in the conduction system. The Naranjo scale is an adverse drug reaction (ADR) probability scale designed to assess drug-related adverse events by utilizing a particular set of questionnaires; it is implemented by some physicians in routine practice to determine whether the cause of the effect is related to a particular drug, which can be helpful for our case.

## 5. Conclusion

All clinicians should be aware of the potential for arrhythmias caused by phenytoin, including those who are on an oral formulation of this drug. Telemetry monitoring can be used in any setting for patients on oral phenytoin with toxic levels or on intravenous phenytoin, even those without evidence of underlying cardiac diseases.
